# Distinct temporal dynamics of judging scene-relative object motion and estimating heading from optic flow

**DOI:** 10.1167/jov.25.13.10

**Published:** 2025-11-18

**Authors:** Mingyang Xie, Li Li

**Affiliations:** 1 Shanghai Key Laboratory of Brain Functional Genomics, School of Psychology and Cognitive Science, East China Normal University, Shanghai, China; 2 New York University-East China Normal University Institute of Brain and Cognitive Science at New York University Shanghai, Shanghai, China; 3 Division of Arts and Sciences, NYU, New York University Shanghai, Shanghai, China

**Keywords:** self-movement, optic flow, object motion, heading, temporal dynamics

## Abstract

During self-movement, the visual system can identify scene-relative object motion via flow parsing and estimate the direction of self-movement (heading) from optic flow. However, the temporal dynamics of these two processes have not been examined and compared using matched displays. In this study, we examined how the accuracy of flow parsing and heading estimation changed with stimulus duration. Participants viewed a stereo optic flow display simulating forward translational self-movement through a cloud composed of wireframe objects with stimulus durations at 100, 200, 400, 700, and 1000 ms. In Experiment 1, a yellow dot probe moved vertically for 100 ms in the scene near the end of the trial. A nulling motion component was added through an adaptive staircase to the probe's image motion to determine when the probe was perceived to move vertically in the scene, which was then used to compute the accuracy of flow parsing. In Experiment 2, participants viewed the same optic flow display without the moving probe object. The simulated heading was randomly varied in each trial, and participants were asked to estimate heading at the end of the trial. As stimulus duration increased, the accuracy of flow parsing decreased, whereas the accuracy of heading estimation increased. These contrasting temporal dynamics suggest that despite both processes relying on optic flow, flow parsing and heading estimation involve distinct processing mechanisms with different temporal characteristics. This divergence, together with previous neurophysiological findings, led us to propose two potential neural mechanisms subserving these two processes to inspire future research.

## Introduction

The ability to precisely perceive one's movement and accurately identify the motion of objects in the environment is vital for effective locomotion and interaction with objects in our surroundings. Optic flow, a concept introduced by Gibson (1950), refers to the pattern of retinal motion generated during self-movement through the environment. This pattern contains important information for perceiving both self-movement and the motion of objects relative to the surroundings, offering key information for navigation. For the perception of self-movement, optic flow allows individuals to identify their direction of self-movement, known as heading (Warren & Hannon, 1988). For example, for straightforward translation, observers tend to use the focus of expansion (FoE) in optic flow to determine their heading (Li, Peli, & Warren, 2002; Warren, Morris, & Kalish, 1988).

For a stationary observer fixating on a point in the scene, identifying scene-relative object motion is straightforward since the object's motion is the only source of retinal motion. However, this task becomes more complex for a moving observer, as the retinal motion of the moving object, in this case, is a combination of optic flow due to self-movement and object motion. Previous research (Warren and Rushton, 2007; Warren and Rushton, 2008; Warren and Rushton, 2009; see also Rushton & Warren, 2005) has proposed that the visual system uses the information in optic flow to remove the self-movement component from the object's retinal motion for the identification of scene-relative object motion, a process known as flow parsing. Many other studies have shown that humans could perform such a computation and estimate scene-relative object motion (e.g., Fajen & Matthis, 2013; Matsumiya & Ando, 2009; MacNeilage, Zhang, DeAngelis, & Angelaki, 2012; Niehorster & Li, 2017; Xie, Niehorster, Lappe, & Li, 2020).

Several studies have examined the relationship between heading perception and flow parsing, identifying both similarities and differences in how these two processes are influenced by visual cues. Foulkes, Rushton, and Warren (2013a) found that both the judgment of heading and the identification of scene-relative object motion during simulated self-movement were influenced similarly by the number of flow vectors and the noise level in optic flow, indicating the coupling between these two processes. This proposal is further supported by the study by Xing and Saunders (2022) that found a trial-by-trial correlation in judgment errors for heading direction and the direction of scene-relative object motion.

On the other hand, several studies have also found evidence supporting the decoupling of heading perception and flow parsing. Warren, Rushton, and Foulkes (2012) provided critical evidence that flow parsing can occur independently from heading estimation. Before this work, it was generally assumed that flow parsing depended directly on the observer first accurately estimating their heading. However, P.A. Warren et al. showed that adding laminar flow to a radial flow field significantly affected observers' heading judgments but did not impair their ability to identify scene-relative object motion. In addition, Rushton, Niehorster, Warren, and Li (2018) reported that form and position cues in optic flow affected heading estimation, whereas only the motion cues in optic flow contributed to the identification of scene-relative object motion. This separation was further supported by the study of Rushton, Chen, and Li (2018), which showed that depth range and gaze rotation influenced heading estimation but not the identification of scene-relative object motion.

Despite these findings, the relationship between heading perception and flow parsing remains largely unknown. Previous research has focused on the effects of various visual cues on these two processes, but to the best of our knowledge, no prior study has examined and compared the temporal dynamics of flow parsing with those of heading estimation using matched displays. Investigating the temporal dynamics of these two processes can provide a comprehensive understanding of their relationship, revealing not only whether they are related but also how they are related over time. This, in turn, can shed light on the underlying neural mechanisms involved in these two processes. The current study aims to address this question.

It has been reported that the visual system can detect motion signals rather quickly. For example, Watson and Turano (1995) found that a simple motion stimulus (such as a moving Gabor patch) lasting 133 milliseconds optimally activated the visual system's motion detectors. However, this quick response does not apply to the processing of optic flow for accurate heading estimation. Specifically, van den Berg (1992) first reported that heading discrimination accuracy declined when the duration of optic flow was reduced to less than 150 ms. Hooge, Beintema, and Van den Berg (1999) examined how quickly observers could locate the FoE and estimate heading from optic flow by tracking their eye movements. Based on the measurements of saccade latency and saccade landing error, they found that finding the FoE in optic flow, on average, took about 430 ms. Layton and Fajen (2016a) examined how the duration of exposure to optic flow affected heading judgments and found that a short exposure duration of 150 ms produced the largest heading bias, which then decreased and stabilized at longer exposure durations around 500 ms.

The relatively long processing time for perceiving heading from optic flow is likely due to the need to not only process the motion information in optic flow but also integrate multiple other visual cues, such as form, position, depth range, and eye rotation, that have been shown to affect heading estimation but not flow parsing (Niehorster, Cheng, & Li, 2010; Rushton et al., 2018; Rushton et al., 2018). Related neurophysiological and brain-imaging studies have identified key brain regions involved in this process. For example, areas such as V3a (Cardin, Hemsworth, & Smith, 2012; Koyama et al., 2005; Kuai et al., 2020; Strong, Silson, Gouws, Morland, & McKeefry, 2017), MST (Britten & van Wezel, 1998; Duffy, 1998; Duffy & Wurtz, 1991; Gu, DeAngelis, & Angelaki, 2012; Peuskens, Sunaert, Dupont, Van Hecke, & Orban, 2001; Schmitt et al., 2020; Tanaka & Saito, 1989), and V3b (Kuai et al., 2020) are tuned to complex optic flow patterns and their activity can be modulated by various non-motion cues that can affect heading estimation. The activation of this broad neural network might explain why perceiving heading from optic flow requires a longer time than processing motion signals.

Given that flow parsing uses motion signals in optic flow to estimate and subtract the self-movement component (Rushton et al., 2018), it might occur rapidly. Indeed, Rushton et al. (2013) reported that flow parsing could occur with exposure time to optic flow as brief as 17 ms. However, no other studies have examined flow parsing at similarly brief durations. More importantly, it remains largely unknown how the accuracy of flow parsing changes with exposure time to optic flow, and how the temporal dynamics of flow parsing compare to those of heading estimation under matched stimulus conditions.

In Experiment 1, we thus examined how the accuracy of flow parsing changes with stimulus duration. The goal is to assess how the exposure time to optic flow affects the subtraction of self-motion component by flow parsing for the judgment of scene-relative object motion during self-movement. Specifically, we used a stereoscopic display to simulate forward observer self-movement to generate optic flow patterns. We varied the stimulus duration to change the exposure time to optic flow. Near the end of the trial, a probe object moved independently in the scene, and we asked observers to judge the moving direction of this probe object at the end of the trial. In Experiment 2, we eliminated the probe object from the scene and asked observers to judge their perceived heading direction at the end of the trial.

By comparing the temporal dynamics of flow parsing and heading estimation, we hope to gain insight into potential differences in how these two perceptual processes use visual information during self-movement. The change in accuracy with exposure time to optic flow for flow parsing versus heading estimation can also shed light on the underlying neural mechanisms subserving these two processes, although definitive conclusions about neural architecture would require complementary neurophysiological evidence. Specifically, a similar pattern of change in accuracy with exposure time to optic flow for both flow parsing and heading estimation may suggest that these two processes rely on a common neural mechanism specialized to process self-movement relevant information. Conversely, distinct patterns of change in accuracy with exposure time to optic flow for these two processes may suggest that flow parsing and heading estimation likely involve separate neural mechanisms. For instance, if flow parsing shows improved accuracy with shorter exposure time, this could reflect an underlying neural mechanism optimized for rapid processing of motion signals in optic flow. In contrast, if heading estimation shows improved accuracy with longer exposure time, this might indicate a neural mechanism that requires extended time to integrate multiple visual cues processed by different neural pathways or circuits.

## Experiment 1: Temporal dynamics of flow parsing

In this experiment, we examined how varying stimulus duration influences the judgment of scene-relative object motion via flow parsing. If flow parsing is independent of heading judgments (Warren et al., 2012) and uses motion signals in optic flow to estimate and subtract the self-movement component without taking other visual cues (such as such as form, position, depth range, and eye rotation) into consideration (Rushton et al., 2018), a short stimulus duration should be sufficient for observers to accurately judge scene-relative object motion. Increasing stimulus duration would not lead to improved accuracy, as the accuracy has already reached the ceiling at shorter stimulus durations.

**Figure 1.**
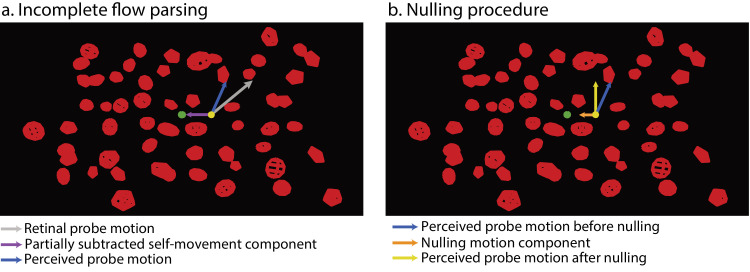
Schematic illustration of (**a**) incomplete flow parsing and (**b**) the nulling procedure. In (**a**), the probe (yellow sphere) moved upward in the scene during simulated forward self-movement, resulting in its retinal motion containing both a component due to self-movement and a component due to its actual motion in the scene (gray arrow line). When the self-movement component (purple arrow line) in the probe's retinal motion is only partially subtracted, the perceived direction of scene-relative probe motion (blue arrow line) deviates rightward from the probe's actual upward direction, indicating incomplete flow parsing. In (b), the orange arrow line indicates the added nulling motion component toward or away from the FOE (green dot) determined by the adaptive staircase procedure to compensate for the residual self-movement component such that the perceived direction of probe motion in the scene (yellow arrow line) reflects its actual upward direction.

To test our hypothesis, we presented participants with a visual scene simulating the observer's forward self-movement where a probe object moved upward in a cloud of wireframe objects (Figure 1). We varied the exposure time to the simulated self-movement and thus optic flow by controlling the stimulus duration time. Participants needed to judge the probe's moving direction in the scene across different stimulus durations. To measure the accuracy of participants’ performance on this task, we used a nulling procedure developed by Niehorster and Li (2017). Specifically, because the visual system has a limited ability to fully compensate for (i.e., subtract) the probe's retinal motion component due to self-movement to recover its actual upward motion in the scene (i.e., incomplete flow parsing), this can lead to a bias in the perceived probe moving direction as illustrated in Figure 1 (see also Layton, Parade, & Fajen, 2023; Niehorster & Li, 2017; Xie et al., 2020). Accordingly, we can compensate for the residual self-movement component in the probe's retinal motion due to incomplete flow parsing by adding a nulling motion component through an adaptive staircase (Kontsevich & Tyler, 1999) until the perceived probe's moving direction in the scene is upward. This gives us a nulling component that can be used to compute the compensation gain, indicating the extent to which the visual system can compensate for the self-movement component in the probe's retinal motion to recover its actual motion in the scene:

(1) $$\begin{array}{c} Gain=\left(1-\frac{v_{n}}{v_{self}}\right)\;*\;100\%,\; \end{array}$$

where *V_self_* is the probe's retinal motion component due to self-movement requiring compensation (subtraction), and *V_n_* is the magnitude of the nulling motion component.

It is important to distinguish between local and global processing of optic flow for the perception of scene-relative object motion. The flow parsing mechanism, as originally proposed (Rushton, Bradshaw, & Warren, 2007; Warren & Rushton, 2009), primarily operates on global flow patterns. Global processing refers to extracting object motion based on the global pattern of optic flow across the visual scene, enabling the visual system to accurately parse out the self-movement component from the object's retinal motion. In contrast, local processing involves identifying object motion based on nearby or localized motion vectors in optic flow directly surrounding the object, which has been found to provide different or complementary information for the perception of scene-relative object motion (e.g., Falconbridge, Hewitt, Haille, Badcock, & Edwards, 2022; Kim, Angelaki, & DeAngelis, 2022; Niehorster & Li, 2017; Royden, Parsons, & Travatello, 2016; Warren & Rushton, 2009).

**Figure 2.**
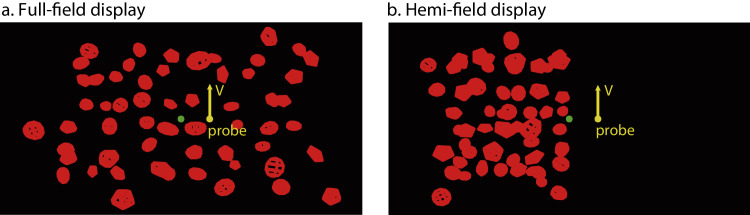
Illustrations of the two display conditions: (**a**) the full-field and (**b**) the hemi-field displays with the probe (yellow sphere) moving vertically upward in the scene at 4° to the right of the fixation point (green dot).

We thus tested two display conditions in the current experiment to manipulate the availability of local versus global processing of optic flow: in (1) the *full-field* display, scene objects covered the entire display and surrounded the probe motion (Figure 2a). Observers could thus rely on both local and global processing of optic flow to judge scene-relative object motion direction. In (2) the *hemi-field* display, the scene object only covered one side of the display opposite to the probe motion (Figure 2b) to limit local motion signals around the probe. This forces observers to rely on global processing of optic flow to judge scene-relative object motion direction through flow parsing. By analyzing changes in the compensation gain across different exposure times to optic flow with these two types of displays, we could examine not only the temporal dynamics of flow parsing but also its interaction with local processing of optic flow.

### Methods

#### Participants

Twelve participants (all naive to the specific goals of the study, four males and eight females) between the ages of 18 and 26 years (mean ± *SD*: 20.83 ± 0.81) participated in this experiment. All had normal or corrected-to-normal vision and provided informed consent approved by the Institutional Review Board at New York University Shanghai. We determined the sample size using power analyses (with power set at 0.8) based on the observed effect sizes from previous similar studies (e.g., Rushton et al., 2018).

#### Visual stimuli and apparatus

The visual display (56°H × 33°V, 120 Hz, viewing distance: 56.5 cm) simulated forward translational self-movement at a typical jogging speed of 2.4 m/s in a three-dimensional (3D) scene that had a typical depth range (5.52–8.24 m) of an artificial environment. Under natural circumstances with scene objects in this depth range, stereo disparity cues would provide information about distance and depth. Therefore we rendered the scene in stereo.

It is difficult for a participant to fuse large, uncrossed disparities on a standard monitor at arm's reach because of the large mismatch with the accommodative or focus cues provided by the surface of the monitor. Therefore we scaled the scene dimensions and the self-movement speed down by a factor of 8. Consequently, the values used to construct the stimuli were a scene-movement speed of 0.3 m/s and a depth range of 0.69 to 1.03 m. The scaling reduced the conflict between the vergence demand and the accommodative demand for near and far objects but kept the angular velocities consistent with those that would be experienced in a 5.52–8.24 m scene approached at 2.4 m/s.

The scene consisted of red wireframe objects moving in a common 3D direction to simulate forward translational self-movement. Each scene object was composed of 5 “slices” and 5 “stacks” (see “gluSphere” in OpenGL Programming Guide, Shreiner, Sellers, Kessenich, & Licea-Kane, 2013) and had an initial radius randomly varied between 1.2–2.7 cm (1.3°–4.5° in visual angle). Two display conditions were tested:

(1) In the *Full-field* condition, we placed 60 scene objects on a rectangular 10 × 6 grid on the image plane before their positions were randomly perturbed (±0.28 cm horizontal and ±0.30 cm vertical) to produce an array of objects that were randomly distributed but not overlapping (Figure 2a). After this placement, the depth of each object was randomly assigned in the entire cloud's depth range (0.69 m to 1.03 m).
(2) In the *Hemi-field* condition, we placed scene objects only on one side of the display, opposite to where the probe was located (Figure 2b). To match the number of visible scene objects (thus a comparable number of motion vectors) between the hemi-field and the full-field display conditions, we used a similar ratio of rows to columns in the grid layout on the image plane as in the full-field condition to place the scene objects. As a result, we placed 56 scene objects on a rectangular 7 × 8 grid on the image plane before their positions were randomly perturbed (±0.24 cm horizontal and ±0.26 cm vertical). After this placement, as in the full-field condition, the depth of each object was randomly assigned in the entire cloud's depth range (0.69 to 1.03 m).

The heading direction of the simulated self-movement was always at the center of the display, indicated by a green fixation dot that appeared throughout the trial. A yellow probe sphere (radius: 0.25 cm) was placed in the mid-depth of the scene and moved upward in a plane perpendicular to the heading direction. The probe movement was shown for 100 ms during the last 200 ms of the simulated self-movement. The midpoint of the probe's movement trajectory was 4° to the left or right of the fixation point. To prevent overlap and ensure clear visibility of the probe, objects located within a 1.5° radius around the probe were removed in the full-field condition. As a result, there were about 56 to 60 scene objects on each trial in the full-field condition.

The probe's speed was chosen such that its vertical instantaneous retinal speed in the middle of the trial was 2°/s. At this eccentricity, the self-movement component in the probe's retinal motion was also 2°/s. A Bayesian adaptive staircase procedure added a horizontal motion component to the probe's retinal motion to find the nulling motion component to compute the compensation gain (see Equation 1).

We tested five exposure times to optic flow corresponding to five stimulus durations of the simulated self-movement: 100, 200, 400, 700, and 1000 ms. For the durations of 200, 400, and 700 ms, the display was created by extracting the last 200, 400, or 700 ms from the 1000 ms display. For the 100 ms display, the time interval used was from 850–950 ms of the 1000 ms display. As such, despite the varying display durations, the mid-point of the probe's movement in each display duration corresponded to the same time point (900 ms) in the 1000 ms display. This consistency maintained an identical depth and speed for the scene-relative probe motion across different stimulus durations, with the only variable being the duration of the display itself.

We rendered the anti-aliased stimuli using MATLAB and the Psychophysics Toolbox 3 on a Leadtek Quadro K2000 GDDR5 graphics card and displayed them on an Asus VG278HE 27″ LCD monitor (ASUS, Taipei, Taiwan) at a resolution of 1920 × 1080 pixels at 120 Hz (60 Hz per eye). Before the experiment began, each participant's cyclopean eye was aligned with the center of the display. With their heads stabilized by a chin rest at the viewing distance of 56.5 cm in a dark room, participants viewed the stimuli through a pair of LCD shutter glasses (Nvidia 3D Vision 2; NVIDIA Corp., Santa Clara, CA, USA) driven by an infrared emitter built into the monitor. Each participant's pupil distance was measured as the input of interocular distance in the program for the generation of the left and right eye images. They were then temporally interleaved and displayed in synchrony with the opening and closing of the left and right eye shutter glass lenses to create a stereoscopic presentation.

#### Procedure

On each trial, the fixation dot first appeared on an empty screen for 1 second. A static view of the scene then appeared for 2 seconds to allow participants to fuse the stereo images into a 3D scene. The scene then moved for 100, 200, 400, 700, or 1000 ms to simulate the forward translational movement of the observer. Participants were asked to keep their gaze on the fixation point throughout the trial. At the end of the motion, a blank screen appeared, and participants pressed the left or right mouse button to indicate whether they perceived the probe moving obliquely leftward or rightward in the scene.

The two display conditions were run in two separate blocks, with their testing order counterbalanced between participants. Each block consisted of 400 randomized trials (40 trials for each staircase × 2 probe locations (left vs. right) × 5 stimulus durations). To familiarize participants with the task, we provided participants with 3-5 practice trials randomly selected from the experimental trials at the beginning of each block. No feedback was provided on any trial. The entire experiment lasted about 45 minutes.

### Results

Given the similar performance for the left and right probe positions for both the full-field and the hemi-field display conditions (main effect of probe position: (*F*(1,11) < 3.78, *p* > 0.078, *η^2^* > 0.26), we combined the data across the left and right probe positions for both display conditions.

**Figure 3.**
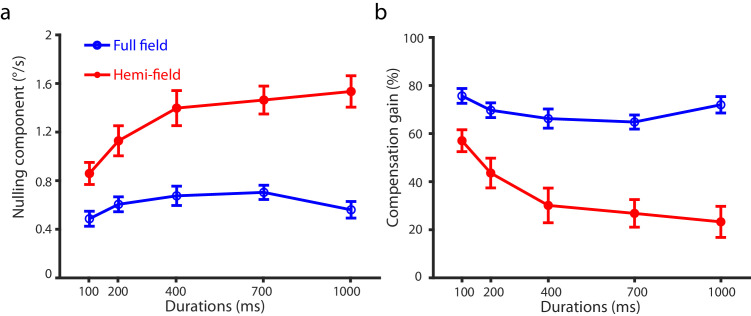
Experiment 1 data. (**a**) Mean nulling motion component and (**b**) mean compensation gain averaged across participants against stimulus duration for the full-field (blue line) and the hemi-field (red line) display conditions. Error bars are ±1 *SE* across 12 participants.

Figure 3 plots the mean nulling component and the mean compensation gain averaged across 12 participants against stimulus duration for the two display conditions. As expected, the nulling component and compensation gain showed contrasting trends, i.e., the larger the nulling component, the smaller the compensation gain. We thus performed statistical analysis on compensation gains only. For both display conditions, compensation gain was significantly larger than zero (*t* (11) > 3.60, *p* < 0.0042, Cohen's *d* > 2.17) and smaller than 100% (*t* (11) < −7.87, *p* < 0.001, Cohen's *d* < −4.75) at all stimulus durations, indicating that participants were able to use information in optic flow to subtract the self-movement component when judging the direction of scene-relative object motion, although the subtraction was not complete.

We conducted a 2 (display condition) × 5 (stimulus duration) repeated-measures analysis of variance (ANOVA) to test the statistical significance of any differences. Across all stimulus durations, compensation gain was lower for the hemi-field than the full-field display condition (main effect of display condition: *F*(1, 11) = 54.98, *p* < 0.001, *η^2^* = 0.83), indicating that participants were more accurate at judging the direction of scene-relative object motion with the full-field than the hemi-field display. Compensation gain also showed a clear difference between stimulus durations (main effect of stimulus duration: *F*(4, 44) = 14.49, *p* < 0.001, *η^2^* = 0.57). Given the significant interaction effect between display condition and stimulus duration (*F*(4, 44) = 11.36, *p* < 0.001, *η^2^* = 0.51), we conducted Newman-Keuls tests and found that while for the hemi-field display condition, compensation gain showed a clear decreasing trend with stimulus duration (34% drop from 100 ms to 1000 ms, *p* < 0.001), for the full-field condition, it decreased primarily between 100 ms and 700 ms (11% drop, *p* = 0.011).

### Discussion

For both display conditions and across all stimulus durations, the larger than zero but smaller than 100% compensation gains indicate that the visual system could indeed use optic flow to parse out the self-movement component from the probe's retinal motion when judging its scene-relative moving direction, but the parsing was incomplete. This is consistent with the findings of previous research studies (e.g., Dokka, MacNeilage, DeAngelis, & Angelaki, 2015; Dupin & Wexler, 2013; MacNeilage et al., 2012; Niehorster & Li, 2017; Warren & Rushton, 2007; Xie et al., 2020) and indicates the limitation of flow parsing relying on visual information from optic flow alone.

The larger compensation gains observed with the full-field than the hemi-field display are also consistent with what was reported by previous studies (e.g., Niehorster & Li, 2017; Warren & Rushton, 2009). That is, relying on both local and global processing of optic flow leads to a more complete removal of the self-movement component in the probe's retinal motion, thus a higher compensation gain and more accurate judgments of the actual probe motion direction in the scene.

The most important finding of this experiment is that for both display conditions, a short exposure time (100 ms) to optic flow resulted in the highest compensation gains, and this is particularly notable for the hemi-field display in which participants could only rely on global processing of optic flow to judge scene-relative object motion through flow parsing. This underscores a crucial aspect of the temporal dynamics of flow parsing and supports our hypothesis that flow parsing uses the motion information in optic flow to estimate and subtract the self-movement component (see also Rushton et al., 2018), and a short exposure time is sufficient for the visual system to extract motion information from optic flow (see also Rushton et al., 2013).

Nevertheless, different from our hypothesis that increasing stimulus duration should not further affect compensation gains because of the ceiling effect, the results show a significant decrease in compensation gain at longer exposure time to optic flow especially for the hemi-field display condition. This trend indicates the visual system's diminishing efficiency in differentiating the retinal motion generated by self-movement from the object's actual motion as stimulus duration increases. We will discuss the possible neural circuits subserving flow parsing that could generate such temporal dynamics in General Discussion.

## Experiment 2: Temporal dynamics of heading estimation

In this experiment, we examined how varying stimulus duration influences the visual system's ability to estimate the direction of heading. In contrast to our hypothesis for flow parsing, we hypothesized that the accuracy of heading estimation would improve with a longer exposure time to optic flow. This is based on previous research studies that have consistently shown that judging heading from optic flow takes between 150 to 430 ms (Hooge et al., 1999; van den Berg, 1992), and the accuracy stabilizes around 500 ms (Layton & Fajen, 2016a). We hypothesize that prolonged exposure time may allow the visual system sufficient time to perform spatial and temporal integration of motion information in optic flow as well as other visual cues, potentially enhancing the accuracy of heading estimation. Because the hemi-field display condition forces observers to rely on global processing of optic flow to judge scene-relative object motion through flow parsing and our goal was to compare the temporal dynamics of flow parsing with those of heading estimation, we tested only the hemi-field display condition in this experiment.

### Methods

#### Participants

Twelve participants (all naive to the specific goals of the study; six males, six females) between the ages of 21 and 27 years (mean ± *SD*: 22.54 ± 0.63) participated in this experiment. All had normal or corrected to normal vision and provided informed consent approved by the Institutional Review Board at the New York University Shanghai. We determined the sample size using power analyses (with power set at 0.8) based on the observed effect sizes from previous similar studies (e.g., Rushton et al., 2018).

#### Visual stimuli and apparatus

We adopted the hemi-field display condition in Experiment 1 and removed the probe object. In addition, the simulated heading direction was randomly varied from −10° (left) to 10° (right) with respect to the center of the display. As in Experiment 1, we tested five stimulus durations of the simulated forward translational self-movement corresponding to five exposure times to optic flow (100, 200, 400, 700, and 1000 ms). The rest of the experimental setup was the same as in Experiment 1.

#### Procedure

On each trial, a green fixation dot (0.19° diameter) appeared at the center of the display for 1 second. A static view of the scene appeared for 2 seconds to allow participants to fuse the stereo images into a 3D scene. The scene then moved for 100, 200, 400, 700, or 1000 ms to simulate the observer's forward self-movement. Participants were asked to keep their gaze on the fixation point throughout the trial. At the end of the trial, a horizontal line appeared at the center of the display. Participants were asked to use a mouse to move a vertical bar (the probe) along the horizontal line to place it in their perceived heading direction.

The experiment consisted of 400 randomized trials (40 trials for each staircase × 2 hemi-field conditions (left vs. right) × 5 durations). To familiarize participants with the task, we provided participants with three to five practice trials randomly selected from the experimental trials at the beginning of the experiment. No feedback was provided on any trial. The entire experiment lasted about 30 minutes.

### Results

**Figure 4.**
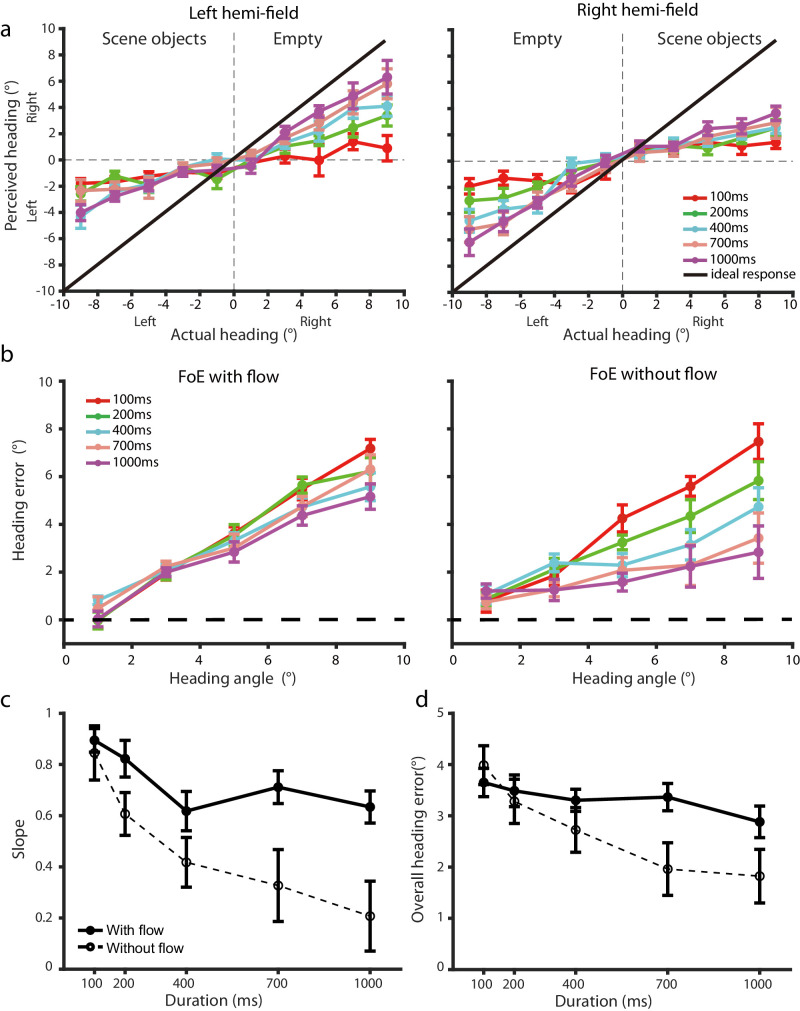
Experiment 2 data. (**a**) Mean perceived heading angle averaged across participants as a function of actual heading angle for the five stimulus durations and the left and right hemi-field displays. The black line represents the perfect performance. (**b**) Mean heading error averaged across participants against actual heading angle for the five stimulus durations and the two types of performance. (**c**) Mean slope of heading error and (**d**) mean overall heading error averaged across heading angles against stimulus duration for the two types of performance. Error bars represent ±1 *SE* across 12 participants.

Figure 4a plots the mean perceived heading angle averaged across participants as a function of the actual heading angle for the five stimulus durations and the left and right hemi-field displays. Positive values indicate heading angles to the right of the center of the display, and negative values indicate heading angles to the left. In both panels, the black line represents the correct response and serves as a reference for ideal performance when the estimated heading perfectly matches the actual heading.

For analysis, we categorized heading performance for the trials in which the simulated heading (i.e., the FoE) was on the same side of scene objects in the hemi-field display as the *FoE-with-flow* performance and for the trials in which the simulated heading was on the empty side of the hemi-field display as the *FoE-without-flow* performance (see Figure 2). Accordingly, for the FoE-with-flow performance, there was background optic flow in the simulated heading direction. Participants could rely on both local and global processing of optic flow for heading estimation. In contrast, for the FoE-without-flow performance, there was no background optic flow in the simulated heading direction. Participants could rely on only global processing of optic flow on the opposite side of the display for heading estimation.

For both the left and right hemi-field displays, as the actual heading deviated from the center of the display, the perceived heading increasingly shifted away from the solid line representing the ideal performance, indicating that participants had a bias of estimating heading toward the center of the display that led to an underestimation of the actual heading angle. However, this center bias in heading estimation decreased as the stimulus duration increased, with a more pronounced reduction in bias observed in the FoE-without-flow performance when there was no background optic flow in the simulated heading direction.

To quantitatively examine the effect of stimulus duration on heading estimation, we calculated heading error, which is defined as the angle between the perceived and the actual heading angle. Positive signs indicate an underestimation of the actual heading angle toward the center of the display, and negative signs indicate an overestimation away from the center of the display. Given the symmetrical performance on the left and right hemi-field displays (see Figure 4a), we combined the heading error data across these two displays for the FoE-with-flow and the FoE-without-flow performance, respectively. Figure 4b plots the mean heading error averaged across participants against actual heading angle for the five stimulus durations and the two types of performance.

For both the FoE-with-flow and the FoE-without-flow performance, the increase in heading error with actual heading angle displayed a clear linear trend. We thus regressed heading error against actual heading angle for each participant's data to obtain the slope of heading error. Any slope larger than zero indicates a center bias in heading estimation. The larger the slope, the larger the bias. Figure 4c plots the mean slope averaged across participants against stimulus duration for both types of performance. We conducted a 2 (performance type) × 5 (stimulus durations) repeated-measures ANOVA to test statistical significance of any differences. Across the two types of performance, slope decreased significantly with stimulus duration (main effect of display duration: *F*(4, 44) = 16.74, *p* < 0.001, *η^2^* = 0.60), indicating a decrease in the center bias with the increase of stimulus duration. Across the five stimulus durations, slope was larger for the FoE-with-flow than the FoE-without-flow performance (main effect of display duration: *F*(1, 11) = 17.52, *p* = 0.001, *η^2^* = 0.61), indicating a larger center bias in heading estimation when there was background optic flow in the simulated heading direction in the display. Given the significant interaction effect of performance type and stimulus duration (*F*(4, 44) = 2.95, *p* = 0.030, *η^2^* = 0.21), we conducted Newman-Keuls tests and found that while the slope for the FoE-without-flow performance showed a clear decreasing trend with stimulus duration (75% drop from 100 ms to 1000 ms, *p* < 0.001), the slope for the FoE-with-flow performance decreased primarily between 100 ms and 400 ms (30% drop, *p* = 0.036).

To examine how the overall accuracy of heading estimation changed with stimulus duration, we averaged heading errors across the tested heading angles to obtain the overall heading error. The smaller the overall heading error, the higher the overall accuracy of heading estimation. Figure 4d plots the mean overall heading error averaged across participants against stimulus duration for both types of performance. We conducted a 2 (performance type) × 5 (stimulus durations) repeated-measures ANOVA to test statistical significance of any differences. Across both types of performance, heading error decreased significantly with stimulus duration (main effect of display duration: *F*(4, 44) = 17.94, *p* < 0.001, *η^2^* = 0.62), indicating an increase in the accuracy of heading estimation with the increase of stimulus duration. Given the significant interaction effect of performance type and stimulus duration (*F*(4, 44) = 7.41, *p* < 0.001, *η^2^* = 0.40), we conducted Newman-Keuls tests and found a significant decrease in heading error from 100 to 1000 ms for both the FoE-with-flow (21% drop, *p* = 0.043) and the FoE-without-flow performance (54%, *p* < 0.001), but such a decrease was larger for the FoE-without-flow than the FoE-with-flow performance as indicated by the significant smaller overall heading error for the FoE-without-flow than the FoE-with-flow performance at both 700 ms (mean ± *SE*: 1.96° ± 0.51° vs. 3.36° ± 0.27°, *p* < 0.001) and 1000 ms (1.82° ± 0.52° vs. 2.88° ± 0.31°, *p* < 0.001).

### Discussion

The observed reduction in the slope of heading error, as well as in the overall heading error with increasing exposure time to optic flow, underscores the temporal dynamics of heading estimation from optic flow and supports our hypothesis that the accuracy of heading estimation improves with longer exposure to optic flow. This result is also consistent with the findings of previous studies showing that the bias in heading judgments decreases with longer exposure to optic flow (Layton & Fajen, 2016a).

Interestingly, our results show that the FoE-with-flow heading judgments displayed a larger center bias than did the FoE-without-flow heading judgments, particularly at longer stimulus durations. This is likely due to the distinct computational demands for heading estimation in these two situations. Specifically, when the simulated heading direction (i.e., the FoE) appeared on the empty side of the hemi-field display with no background optic flow (i.e., FoE-wihout-flow), participants could estimate the location of the FoE by extrapolating from available motion vectors on the opposite side. As shown by Li et al., (2002), when the FoE lies outside the visible field, observers estimate its location by determining the intersection point of at least two motion vectors. However, errors can arise in this triangulation process due to noise in extracting the direction of the motion vectors, which increase as motion vectors are sampled farther from the FoE (Koenderink & van Doorn, 1987). The resulting triangulation errors are asymmetrical, often displacing the estimated FoE away from the center of the display (see Figure 6 in Li et al., 2002). This displacement effectively counteracts the center bias typically observed in heading judgments and, paradoxically, leads to reduced heading errors in the FoE-without-flow heading judgments, whereas the FoE-with-flow heading judgments remain subject to the usual center bias effect.

Note that even for the FoE-with-flow heading judgments at the longest stimulus duration of 1000 ms, the mean slope of heading error is still at about 0.6 with the mean overall heading error of about 3°. This level of accuracy in heading estimation is not as high as what has been reported (1-2°) by previous studies (e.g., Foulkes, Rushton, & Warren, 2013b; Li et al., 2002; van den Berg, 1992; Warren et al., 1988). One possible explanation could be that the hemi-field display provided optic flow only in half of the visual field. In addition, participants were asked to fixate on a fixation point in the center of the display throughout the trial, which might have increased the center bias effect in heading estimation and led to larger heading errors.

The most important finding of the current experiment is that the temporal dynamics of heading estimation differ markedly from those of flow parsing observed in Experiment 1. In Experiment 1, the accuracy of flow parsing decreased with increasing stimulus duration in the hemi-field display condition. In Experiment 2, the accuracy of heading estimation showed consistent improvement with longer stimulus durations regardless of whether the simulated heading direction appeared in the empty or the scene-object side of the hemi-field display. These contrasting findings from the two experiments reveal the distinct temporal dynamics of flow parsing and heading estimation despite the fact that both rely on optic flow and can be influenced by the number of flow vectors and the noise level in optic flow (Foulkes et al., 2013a). We discuss the implications of these findings in the next section.

## General discussion

The results of the two experiments show that for the hemi-field display in which the contribution of local processing of optic flow to the perception of scene-relative object motion is removed, the accuracy of flow parsing decreases with exposure time to optic flow. In contrast, the accuracy of heading estimation increases with exposure time to optic flow. Whereas it takes as short as 100-ms exposure to optic flow for flow parsing to reach the highest accuracy, it takes much longer exposure to optic flow for accurate heading estimation to happen. This contrasting trend of the temporal dynamics of flow parsing and heading estimation shows a clear decoupling between these two processes.

Xing and Saunders (2022) reported that flow parsing and heading estimation showed a trial-by-trial correlation in judgment errors and thus were coupled. In their study, on each trial, participants viewed the displays that simulated forward self-movement for 1 s before an independently moving object appeared and remained visible for 1 s. Participants were then asked to report the object's motion direction and their perceived heading direction. Given that an independently moving object induces a small but systematic bias in heading judgments (e.g., Layton & Fajen, 2016a; Layton & Fajen, 2016b; Li, Ni, Lappe, Niehorster, & Sun, 2018; Royden & Hildreth, 1996; Warren & Saunders, 1995), heading judgments in their study could be affected by the moving object, leading to the observed correlation in judgment errors. Accordingly, their results are inconclusive regarding the relationship between heading perception and flow parsing.

Although the visual system can accurately identify an independently moving object in the flow field during self-movement (e.g., Niehorster & Li, 2017; Rushton & Warren, 2005; Warren & Rushton, 2009), heading estimation in this case does not segregate the moving object out and is affected by the object motion (Layton & Fajen, 2016b; Li et al., 2018; Royden & Hildreth, 1996; Warren & Saunders, 1995). These findings suggest that flow parsing and heading estimation do not depend on teach other. Together with the previous findings showing that flow parsing can indeed occur independently from heading estimation (Warren, Rushton, & Foulkes, 2012) and the observed divergence in temporal dynamics between these two processes in the current study, we propose that although the perception of heading and the perception of scene-relative object movement during self-movement both rely on information from optic flow, they likely involve distinct processing mechanisms with different temporal characteristics.

Below, we review current findings regarding cortical areas involved in heading estimation and flow parsing. We then combine the findings of our study with previous neurophysiological findings to propose two possible neural mechanisms that involve different brain areas and in different ways for flow parsing and heading estimation.

### Cortical areas involved in heading estimation and flow parsing

Mishkin and Ungerleider (1982) have long proposed that visual information is first processed in the striate visual cortex (area V1) and then passed to the extrastriate visual cortex along two separate visual pathways: The dorsal pathway to the posterior parietal cortex is in charge of visuospatial and motion perception, and the ventral pathway to the inferior temporal cortex is in charge of object perception. This separation can begin as early as the subcortical lateral geniculate nucleus (LGN) in thalamus, i.e., the parvocellular (P) layers of the LGN projecting mainly to the ventral pathway show a high sensitivity to color and spatial resolution related to object fine details, whereas the magnocellular (M) layers of the LGN projecting primarily to the dorsal pathway show a high sensitivity to contrast and temporal resolution related to object motion (for a review, see Livingstone & Hubel, 1988).

It has long been proposed that optic flow is processed primarily by area MST along the dorsal pathway. Previous neurophysiological studies on non-human primates have shown that area MST receives inputs from and also sends feedback signals to area MT that is responsive to motion signals (Maunsell & Van Essen, 1983). Neurons in MST have receptive fields much larger than those in MT and respond to optic-flow stimuli (Duffy, 1998; Duffy & Wurtz, 1991; Saito et al., 1986; Tanaka & Saito, 1989). Using electrical microstimulation, Britten and van Wezel (1998) found that stimulations to neurons in MST affected heading judgments in monkeys. The dorsal part of MST (MSTd) has also been shown to be in charge of integrating multisensory information (such as visual and vestibular information) for heading estimation (Gu et al., 2012). Although these findings are based on non-human primates, substantial evidence suggests the existence of an anatomically and functionally homologous MST area in humans (for a review, Wild & Treue, 2021).

The human homolog of the non-human primate MST is located within the MT+/V5 complex, with hMST positioned in the anterior part that responds to contra- and ipsilateral optic flow and hMT in the posterior part that responds to contralateral motion stimuli (Dukelow et al., 2001; Huk, Dougherty, & Heeger, 2002; Smith, Wall, Williams, & Singh, 2006). Using fMRI, Wall, Lingnau, Ashida, and Smith (2008) found that hMST is sensitive to rotation and expansion optic flow. Smith, Wall, and Thilo (2012) combined galvanic vestibular stimulation and fMRI technique and found that hMST also responded to vestibular information about self-movement.

Research has shown that MT+ plays a crucial role in heading estimation from optic flow in humans. For example, Peuskens et al. (2001) used PET and fMRI to investigate brain activation when participants viewed stimuli depicting translational self-movement over a ground plane with no shift in the FoE location or with the FoE shifted leftward and rightward in a sinusoidal manner simulating changes in heading direction. Participants were asked to judge the shifting direction of the FoE. They found that MT+ and the dorsal intraparietal sulcus (DIPSM/L) were specifically activated when performing this task. Recently, Schmitt et al. (2020) asked participants to view optic flow stimuli depicting self-movement over a ground plane and report their perceived heading. They found that disrupting hMST with TMS increased the variance in heading estimation, providing causal evidence for the role of hMST in heading estimation from optic flow.

Other cortical areas that have been shown to be involved in heading estimation include VIP and CSv. However, the roles of these two areas are still under debate. Specifically, several studies have found that VIP neurons in macaque monkeys are tuned to the FoE location in optic flow (e.g., Bremmer, Duhamel, Ben Hamed, & Graf, 2002; Zhang and Britten, 2004). Zhang and Britten (2011) also reported that electrically stimulating VIP neurons biased monkeys' judgments of heading toward the preferred direction of the stimulated neuron. However, more recent studies, such as Chen, Gu, Liu, DeAngelis, and Angelaki (2016), challenged this view by showing that bilateral suppression of VIP in macaques had no effect on heading judgments. Although hMST can be probed using TMS to explore its causal role in heading estimation, VIP's deep location in the human brain makes it inaccessible to TMS. This limits the ability to conclusively determine whether VIP is causally involved in heading estimation in humans.

Research on CSv consistently highlights its response to changes in heading during self-movement. Initial human brain-imaging studies by Wall and Smith (2008) reported that CSv responded much more strongly to combined expansion and rotation flow patterns, than simple expansion patterns indicating a fixed heading direction. Furlan, Wann, and Smith (2014) directly compared the brain's responses to optic flow depicting forward self-movement with a constant heading or with a sinusoidal lateral component that caused heading to change continuously. CSv's response was four times larger for stimuli depicting a changing heading than a constant heading, a much more pronounced difference than seen in other visual areas. Nevertheless, the stimuli depicting a changing heading in these studies were confounded with the change in the type of flow patterns (e.g., expansion vs. expansion + rotation), making it unclear whether CSv is specialized for detecting heading changes per se, or merely responding to changes in the overall optic flow pattern.

Research findings from many studies provide converging evidence that MT+ also plays an important role in flow parsing. Calabro and Vaina (2012) conducted the initial fMRI study to identify human brain regions involved in flow parsing. Participants viewed displays depicting simulated forward self-movement with nine textured objects embedded in the scene, and their task was to identify the target object that moved toward or away from the observer. They found that occipito-temporal areas, including MT+, along with V1, V2, LO, V3a, and KO (V3b), were activated when performing this task. Using the same task, Kozhemiako et al. (2020) conducted a MEG study with a Support Vector Classifier (SVC) to predict task performance. Their findings further confirmed the role of MT+ in flow parsing. Other later fMRI studies found that regions like V3a, MT+, and V6 were all involved in distinguishing between stimuli depicting self-movement and object motion (Pitzalis et al., 2020; Sulpizio et al., 2024). A recent fMRI study from our lab used two types of visual stimuli with identical flow fields, but one contained scene-relative object motion, and the other did not. Using multi-voxel pattern analysis, we found that the decoding accuracy for these two types of stimuli was significantly above the chance level in V7 and MT+, offering compelling evidence of MT+ playing a critical role in flow parsing (Shen, Shan, Rushton, Kuai, & Li, 2024).

### Possible neural mechanisms for heading estimation and flow parsing

**Figure 5.**
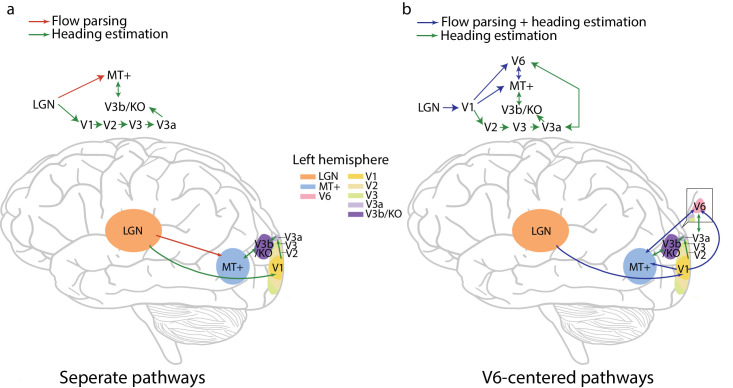
Possible neural mechanisms for heading estimation and flow parsing. (**a**) A direct and fast visual pathway for flow parsing and a slower pathway for heading estimation. (**b**) area V6-centered pathways for flow parsing and heading estimation with the latter involving additional brain areas and reactivation of V6. Adapted from Li (2025).

The distinct temporal dynamics of flow parsing and heading estimation observed in the current study along with previous neurophysiological findings prompted us to propose two possible neural mechanisms subserving these two processes: (1) separate pathways with flow parsing benefiting from a rapid, direct pathway and heading estimation requiring a slower pathway for multi-area integration of additional cues like form, position, and depth range (Figure 5a), and (2) area V6-centered pathways that support both functions through activation and reactivation of V6 (Figure 5b). This section examines the evidence for these two neural mechanisms.

#### Separate pathways

Azzopardi, Fallah, Gross, and Rodman (2003) recorded neuron firings in the macaque brain and found that the response of MST neurons to both slow and fast motion showed little change even after unilateral V1 lesions, indicating that motion information can reach this area bypassing V1. Sincich, Park, Wohlgemuth, and Horton (2004) used retrograde tracing techniques and immunostaining and found that there is a direct projection from the LGN to MT.

Much research on humans also supports a direct visual pathway from the LGN to MT+ that bypasses V1. Specifically, Ffytche, Guy, and Zeki (1995) combined the high temporal resolution of EEG and MEG with the precise spatial data from PET to explore the parallel processing of visual motion inputs in V1 and V5. They found that activity peaked earlier in MT+ than V1 for fast motion. Gaglianese, Costagli, Bernardi, Ricciardi, and Pietrini (2012) reported that activity in the LGN directly affected activity in MT+ independent of V1. Using Conditional Granger Causality analysis, they later found a direct pathway carrying motion information from thalamus to MT+ (Gaglianese et al., 2015).

Behaviorally, Azzopardi and Hock (2011) tested a cortically blind (damage to V1) man using the reverse-phi illusion and found that he was still able to perceive motion in his blindsight based on the first-order motion energy (spatiotemporal changes in luminance). They thus proposed two functionally different visual pathways for processing motion signals from the retina to MT+: one passes through V1 and specializes in feature-based motion perception, and the other bypasses V1 and is dedicated to detecting motion energy. Pelah, Barbur, Thurrell, and Hock (2015) later examined a patient who had a V1 lesion in the left hemisphere and found that optic flow could still be processed in time in the same hemisphere, supporting the existence of visual pathways for processing optic flow that bypass V1. This view is further supported by Ajina and Bridge (2018), who used fMRI and found that blindsight depends on a functional connection between the LGN and MT+, highlighting the critical role of the LGN–MT+ pathway in motion perception following V1 damage.

Given that flow parsing is a fast process and uses motion signals in optic flow to estimate and subtract the self-movement component (Rushton et al., 2018), optic flow could be quickly processed from the LGN to MT+, bypassing V1 for the identification of scene-relative object motion during self-movement (Figure 5a, see also Rushton et al., 2018). In the meantime, due to the fact that heading estimation can be affected by not only motion but many other visual cues such as form, position, depth range, and eye rotation (Niehorster et al., 2010; Rushton et al., 2018; Rushton et al., 2018), more brain areas might be involved in heading estimation, leading to a longer processing time than flow parsing. For example, neurons in macaque area V3a exhibit selectivity for translational motion as well as complex optic flow patterns (Nakhla, Korkian, Krause, & Pack, 2021). In the human brain, V3a has been shown to respond to both translational and radial flow patterns (Cardin et al., 2012; Koyama et al., 2005). Strong et al. (2017) used TMS to disrupt V3a activity and found that it impaired participants' ability to judge the location shift in the FoE in radial optic flow. Kuai et al. (2020) used fMRI and found that activities in V2, V3d, V3a, and V3b/KO can all be modulated by the change in location of the FoE defined by either motion or form cues in radial flow patterns. Accordingly, optic flow could be initially processed in V1 and then passed through areas (such as V2, V3d, V3a, and V3b/KO) for the processing of other visual cues. The integration of all the neural signals happens in MT+ for the estimation of heading (Figure 5a).

The neural mechanism with two separate visual pathways for flow parsing and heading estimation proposed above can explain not only the distinct temporal dynamics of these two processes observed in the current study, but also why both flow parsing and heading perception can be affected by the number of motion vectors and the noise level in optic flow (Foulkes et al., 2013a), but various visual cues (such as form, position, and depth range) can affect heading estimation not flow parsing (Rushton et al., 2018; Rushton et al., 2018; Warren et al., 2012). There is also a possibility that MT+ works on flow parsing computation in the early stage but splits off to perform primarily heading computation in a later stage, leading to a decreased accuracy in flow parsing but an increased accuracy in heading estimation with time, as observed in the current study.

#### V6-centered pathways

In the past two decades, another brain area that has been identified to play an important role in the perception of self-movement is area V6 (Fan, Liu, DeAngelis, & Angelaki, 2015), located in the parieto-occipital sulcus of the macaque and the human brain (Galletti, Battaglini, & Fattori, 1991; Galletti, Fattori, Battaglini, Shipp, & Zeki, 1996; Galletti, Fattori, Gamberini, & Kutz, 1999; Pitzalis et al., 2006; Pitzalis et al., 2010). Considering the findings of our current study that flow parsing is a rapid process and heading estimation is a relatively slower process, the processing temporal dynamics in V6 could provide us with another possibility for the underlying neural mechanism.

Pitzalis et al. (2010) first identified the human homolog of V6 and showed that V6 responded to the coherent but not incoherent motion of random-dot patterns. Cardin and Smith (2010) conducted an fMRI study in which participants viewed expanding dot patterns with stereoscopic depth cues that were either consistent or inconsistent with self-movement. They found that V6 responded strongly to consistent combinations, suggesting that V6 integrates depth and motion cues in optic flow for the perception of self-movement. Several other brain-imaging studies found that V6 responded to not just optic flow (Cardin, Sherrington, Hemsworth, & Smith, 2012; Pitzalis et al., 2013; Pitzalis et al., 2013) but more strongly to optic flow stimuli depicting self-movement along a curved path with changing heading rather than a straight path with constant heading (Di Marco et al., 2021; Furlan et al., 2014). More recently, Pitzalis et al. (2020) found that V6 also responded to scene-relative object motion with background optic flow. These findings suggest that V6 is involved in processing optic flow for flow parsing and heading estimation.

Importantly, V6 not only responds to motion stimuli, but the response happens fast. Specifically, von Pföstl et al. (2009) recorded neuromagnetic signals and found that the response of V6 to drifting gratings occurred at approximately 110 ms from the stimulus onset. Combining visual evoked potentials with fMRI, Pitzalis et al. (2013) observed that the response of V6 to combined spiral and radial flow patterns was almost simultaneous with that of MT+, i.e., V1 responded at about 50 ms with the peak response time at 75 ms. MT+ and V6 showed similar onset latencies only a bit later (100 ms and 105 ms, respectively) with the peak response time at 130 ms and 140 ms, respectively. This suggests a parallel and fast visual motion stream from V1 to V6, which could serve the fast flow parsing process.

Interestingly, Pitzalis et al. (2013) also found a second activation of V6 at approximately 230 ms. They proposed that the second activation in V6 could be a re-entrant feedback from other extrastriate visual areas, particularly V3a. The connection between V3a and V6 has been established in studies involving both macaque monkeys (Galletti et al., 2001) and humans (Tosoni et al., 2015). We propose that the second activation observed by Pitzalis et al. (2013) could serve the relatively slower heading estimation process (i.e., other heading informational cues are processed by V1 and other extrastriate visual areas in sequence, then all the neural signals arrive at V6 and activate it again for heading estimation).

Taking the processing time dynamics in V6 into consideration, we thus propose that motion cues in optic flow are processed and then delivered directly from V1 to MT+ and V6, which can be used for fast flow parsing. In the meanwhile, other visual cues that can affect or be used for heading estimation are processed and delivered from V1 to other extrastriate areas (such as V2, V3, V3a, and V3b/KO), which then reactivate V6 or MT+ for the combination with motion cues for heading estimation (see Figure 5b). As a result, heading estimation takes longer to become accurate due to the involvement and processing time of all these areas. Furthermore, MT+ and V6 could be primarily involved in flow parsing at the beginning but become more involved in heading estimation as neural signals of the other cues come in, leading to decreased flow parsing and increased heading estimation accuracy with stimulus duration.

## Conclusions

In this study, we examined the temporal dynamics of judging scene-relative object motion through flow parsing and estimating heading from optic flow. Our results reveal distinct temporal dynamics of these two processes. That is, with the hemi-field display that forces observers to rely on global processing of optic flow to judge scene-relative object motion via flow parsing, the accuracy of flow parsing decreases with longer stimulus durations. In contrast, the accuracy of heading estimation consistently increases with stimulus duration. This divergence, along with previous neurophysiological findings, led us to propose two possible neural mechanisms subserving these two processes.

First, we propose that functional separation may begin as early as area V1, with flow parsing bypassing V1, while heading estimation involves V1 and other extrastriate visual areas. Alternatively, it is possible that both flow parsing and heading estimation are processed primarily in areas V6 and MT+, with heading estimation requiring additional cortical areas to integrate multiple visual cues that do not affect flow parsing. We hope these hypotheses will inspire future research to investigate neural mechanisms responsible for flow parsing and heading estimation.
